# Advances in physicochemical characterization of lead-free hybrid perovskite [NH_3_(CH_2_)_3_NH_3_]CuBr_4_ crystals

**DOI:** 10.1038/s41598-022-12832-y

**Published:** 2022-05-24

**Authors:** Ae Ran Lim, Lee Ku Kwac

**Affiliations:** 1 Graduate School of Carbon Convergence Engineering, Jeonju University, Jeonju, 55069 Korea; 2 Department of Science Education, Jeonju University, Jeonju, 55069 Korea; 3 Institute of Carbon Technology, Jeonju University, Jeonju, 55069 Korea

**Keywords:** Chemistry, Materials science

## Abstract

To support the development of eco-friendly hybrid perovskite solar cells, structural, thermal, and physical properties of the lead-free hybrid perovskite [NH_3_(CH_2_)_3_NH_3_]CuBr_4_ were investigated using X-ray diffraction (XRD), differential scanning calorimetry, thermogravimetric analysis, and nuclear magnetic resonance spectroscopy. The crystal structure confirmed by XRD was monoclinic, and thermodynamic stability was observed at approximately 500 K without any phase transition. The large changes in the ^1^H chemical shifts of NH_3_ and those in C2 close to N are affected by N–H∙∙∙Br hydrogen bonds because the structural geometry of CuBr_4_ changed significantly. The ^1^H and ^13^C spin–lattice relaxation times (T_1ρ_) showed very similar molecular motions according to the Bloembergen–Purcell–Pound theory at low temperatures; however, the ^1^H T_1ρ_ values representing energy transfer were about 10 times lesser than those of ^13^C T_1ρ_. Finally, the ^1^H and ^13^C T_1ρ_ values of [NH_3_(CH_2_)_3_NH_3_]*Me*Br_4_ (*Me* = Cu, Zn, and Cd) were compared with those reported previously. ^1^H T_1ρ_ was affected by the paramagnetic ion of the anion, while ^13^C T_1ρ_ was affected by the *Me*Br_4_ structure of the anion; ^13^C T_1ρ_ values in *Me* = Cu and Cd with the octahedral *Me*Br_6_ structure had longer values than those in *Me* = Zn with the tetrahedral *Me*Br_4_ structure. We believe that these detailed insights on the physical properties will play a crucial role in the development of eco-friendly hybrid perovskite solar cells.

## Introduction

**Figure 1:**
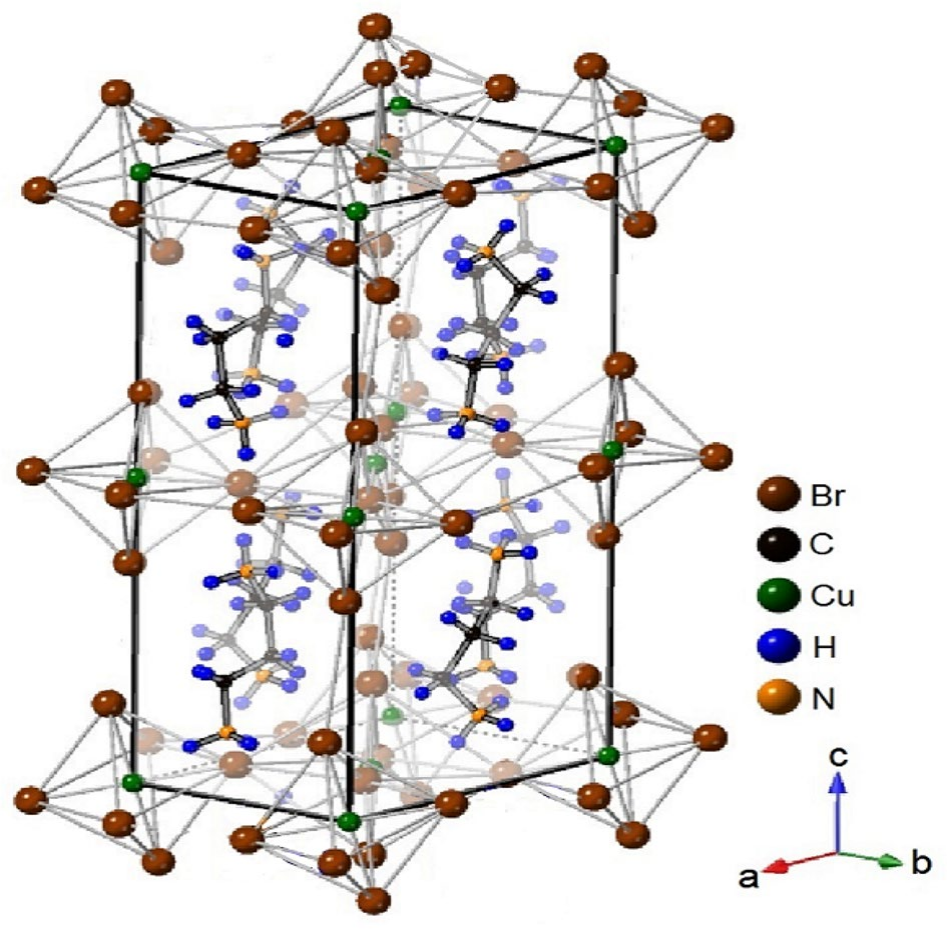
Crystal structure of [NH_3_(CH_2_)_3_NH_3_]CuBr_4_ (CCDC: 1278590).

The development of solar cells based on CH_3_NH_3_Pb*X*_3_ (*X* = Cl, Br, and I) type organic–inorganic hybrid materials have advanced recently. However, due to the presence of Pb, these perovskites decompose in humid air and are toxic. Therefore, the development of alternative eco-friendly hybrid perovskite solar cells is crucial^1–5^. The synthesis of novel groups of organic–inorganic materials, as well as improved functional materials, has attracted significant attention. The fabrication of hybrid perovskites has recently been reported as a major challenge in the context of developing ferroelastic semiconductors^6^. On the other hand, the success of single-crystal-level ferroelectric performance makes hybrid perovskites suitable candidates for flexible and wearable devices^7,8^. As an eco-friendly alternative to sunlight, the need for detailed characterization of perovskite structures and dynamics of the new organic–inorganic hybrid compounds [NH_3_(CH_2_)_*n*_NH_3_]*MeX*_4_ (*Me* = Mn, Fe, Co, Cu, and Cd, *n* = 2, 3 …) with various configurations is increasing in relation to their potential applications in photovoltaic performance^9–18^. Perovskites comprising [NH_3_(CH_2_)_*n*_NH_3_] and *MeX*_4_-layered metal-halogen anionic sublattices are an interesting group of hybrid materials^16,19–25^. Their physicochemical properties are related to their structure and the interaction between the organic and inorganic components. The organic cations are related to the structural flexibility and optical properties, and the inorganic anions are related to the thermal and mechanical properties^26^. For *Me* = Mn, Cu, or Cd, the structure is two-dimensional and comprises a corner-shared octahedral (*MeX*_6_)^2−^ with alternating organic layers. When *Me* = Co or Zn, the structures are tetrahedral (*MeX*_4_)^2−^ groups sandwiched between layers of organic cations and are zero-dimensional^9,16,20,27^. In [NH_3_(CH_2_)_*n*_NH_3_]*MeX*_4_, the N–H···*X* hydrogen bonding occurs between the NH_3_ groups at both ends of the organic chains and *X* group of the perovskite-type layer. Among these materials, [NH_3_(CH_2_)_3_NH_3_]CuBr_4_ [bis (propylene-1, 3-diammonium) tetrabromocuprate] with *n* = 3, *Me* = Cu, and *X* = Br has a monoclinic structure with the *P2*_*1*_*/n* space group at room temperature. The unit cell dimensions are *a* = 8.086 Å, *b* = 7.566 Å, *c* = 17.622 Å, β = 96.75°, and Z = 4^28^. The structural geometry of [NH_3_(CH_2_)_3_NH_3_]CuBr_4_ is shown in Fig. 1 (CCDC: 1278590)^28^. The structure is composed of puckered layers of CuBr_6_ octahedra separated by layers of [NH_3_(CH_2_)_3_NH_3_]^2+^ chains that are nearly perpendicular to the layers. At both ends of the cation, ammonium groups were located between the layers. Extensive N–H···Br type hydrogen bonding was found between the Cu and Br layers and the alkylammonium chain. The organic chains extended along the crystallographic c-direction.

Several reports have been published on related materials. For example, Snively et al.^29^ reported the two-halide linear super-exchange bridge of the [NH_3_(CH_2_)_3_NH_3_]CuBr_4_ crystal, and Straatman et al.^30^ discussed the theoretical analysis of double-halide super-exchange. Kite and Drumheller^31^ conducted an electron paramagnetic resonance study of this crystal in a temperature range below the room temperature. Kallel et al.^32^ determined structure of [NH_3_(CH_2_)_3_NH_3_]ZnBr_4_ for *Me*=Zn by X-ray diffraction (XRD) analysis. The [NH_3_(CH_2_)_3_NH_3_]CdBr_4_ crystal structure for *Me*=Cd has been reported by XRD analysis at room temperature, and the temperature dependence of the ^79,81^Br nuclear quadrupole resonance spectrum near the phase transition temperatures was studied by Ishihara et al.^33,34^ Recently, our group reported the results for [NH_3_(CH_2_)_3_NH_3_]*Me*Br_4_ (*Me*=Zn and Cd) single crystal studies^27,35^; physicochemical properties and structural dynamics were mainly studied from the chemical shifts and spin-lattice relaxation times using nuclear magnetic resonance (NMR).

The structure and lattice constant of the [NH_3_(CH_2_)_3_NH_3_]CuBr_4_ single crystal grown in this study was confirmed by XRD. To understand the role of inorganic anions on the thermodynamic properties, differential scanning calorimetry (DSC) and thermogravimetric analysis (TGA) experiments were performed. In addition, the role of the organic cation in the structural properties was considered in detail using the magic angle spinning (MAS) NMR method. The chemical shifts and spin-lattice relaxation times T_1ρ_ are discussed for ^1^H and ^13^C. In particular, the N–H···Br hydrogen bond between the Cu and Br layer and the cation within [NH_3_(CH_2_)_3_NH_3_]CuBr_4_ is expected to provide important characteristics for the development of perovskite material based solar cells. Finally, the physical properties of [NH_3_(CH_2_)_3_NH_3_]*Me*Br_4_ (*Me*=Cu, Zn, and Cd) crystals were compared with the previous reports and explained based on the paramagnetic ion and the structure of the *MeX*_4_ anion.

## Methods

### Materials

[NH_3_(CH_2_)_3_NH_3_]CuBr_4_ crystals were prepared with molecular weights of NH_2_(CH_2_)_3_NH_2_·2HBr and CuBr_2_ with 1:1 ratio in an aqueous solution. The mixture was stirred and heated to obtain a homogeneous solution. The resulting solution was filtered and brown colored single crystals were obtained by slow evaporation over few weeks. The crystals grew into rectangular shapes with dimensions of 5 × 5 × 1 mm^3^.

### Characterization

The structure of the [NH_3_(CH_2_)_3_NH_3_]CuBr_4_ crystal at 298 K was determined by single-crystal XRD system (Bruker D8 Venture, Germany) with Mo-Kα radiation at the Western Seoul Center of the Korea Basic Science Institute (KBSI). DSC (TA Instruments, DSC 25, USA) was performed at a heating rate of 10 °C/min in the 190-600 K temperature range in a N_2_ gas atmosphere. Thermogravimetric analysis (TGA) was conducted using a thermogravimetric analyzer (TA Instruments, USA) in the 300–680 K temperature range at the same heating rate. Optical observations were performed using a Carl Zeiss microscope equipped with a Linkam THM-600 heating stage.

NMR spectra of the [NH_3_(CH_2_)_3_NH_3_]CuBr_4_ crystals were collected on a Bruker Avance II+ NMR spectrometer at the same KBSI center. The Larmor frequencies for the ^1^H NMR and ^13^C NMR experiments were 400.13 and 100.61 MHz, respectively. NMR chemical shifts were referenced to tetramethylsilane (TMS) as the standard. The powdered sample in the cylindrical zirconia rotor was spun at a rate of 10 kHz in the MAS NMR experiment. The spin-locking field during the ^1^H and ^13^C NMR acquisition was 71.42 kHz. The ^1^H T_1ρ_ and ^13^C T_1ρ_ values were measured by changing the spin-locking pulse duration. ^1^H and ^13^C NMR data were measured using a single pulse sequence with a pulse width of 3.25–3.59 μs. NMR data could not be measured at temperatures above 430 K because NMR equipment that could measure at such high temperatures are unavailable.

## Experimental results

### Crystal structure

Single-crystal XRD analysis of [NH_3_(CH_2_)_3_NH_3_]CuBr_4_ was performed at 298 K. The structure, lattice constants, and space group of this crystal were monoclinic, with *a* = 8.052 ± 0.009 Å, *b* = 7.560 ± 0.009 Å, *c* = 17.611 ± 0.190 Å, β = 96.920 ± 0.05°, Z = 4, and *P*2_1_/*n*, and this result was in good agreement with a previous report by Halvorson and Willett^28^.

### Thermodynamic property

**Figure 2:**
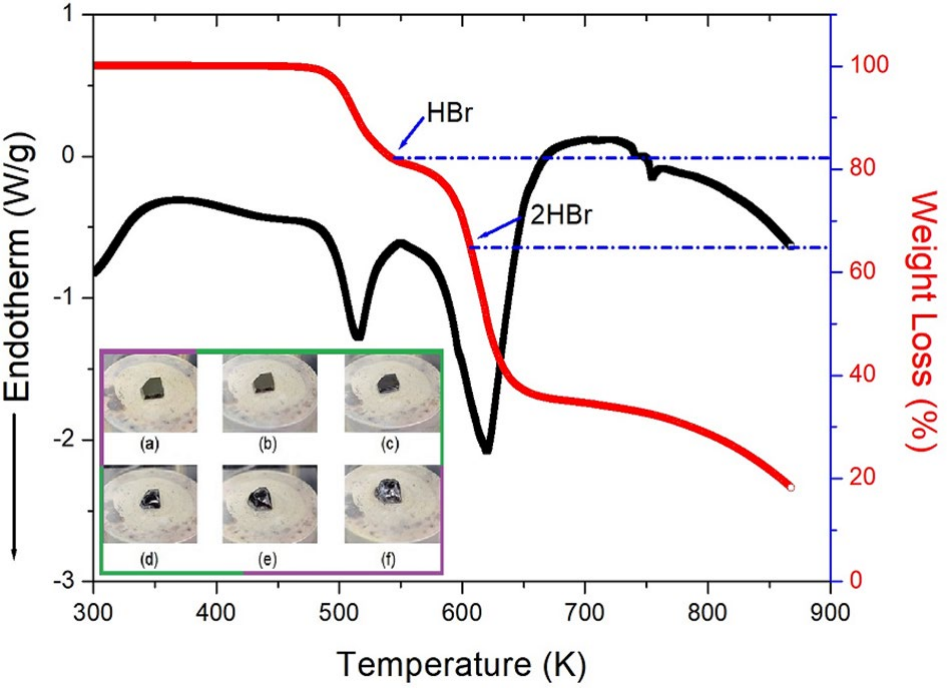
Thermogravimetric analysis (TGA) and differential thermal analysis (DTA) curves of [NH_3_(CH_2_)_3_NH_3_]CuBr_4_ (inset: Changes in crystal measured by optical polarizing microscopy at (**a**) 300, (**b**) 453, (**c**) 500, (**d**) 543, (**e**) 593, and (**f**) 621 K).

In the DSC experiment, there was no phase transition temperature in the 200–500 K range; however, large exothermic peaks were observed at 546 and 577 K (Supplementary Information 1). To confirm that the DSC peaks at 546 and 577 K are related to the phase transition, TGA and differential thermal analysis (DTA) experiments were performed; the results are shown in Fig. 2. The TGA results revealed that this crystal is thermally stable up to 504 K. The initial weight loss of [NH_3_(CH_2_)_3_NH_3_]CuBr_4_ began at 504 K, and there was no weight loss before the decomposition temperature. In the TGA curve, [NH_3_(CH_2_)_3_NH_3_]CuBr_4_ exhibited a two-stage decomposition at high temperatures. The initial weight loss (17%) occurred in the 500–550 K range, which may be due to the decomposition of HBr in [NH_3_(CH_2_)_3_NH_3_]CuBr_4_. The second-stage decomposition (63%) occurred because of the presence of an inorganic moiety in the 550–650 K range. The amount that remained as a solid was calculated from the TGA data and chemical reactions. The weight losses of 17 and 35% at approximately 546 and 607 K are likely due to the decomposition of the HBr and 2HBr moieties, respectively, which is consistent with the exothermic peak in the DSC curve. The molecular weight decreased abruptly between 550 and 650 K, with a corresponding weight loss of 63% at approximately 650 K.

Further, optical polarizing microscopy experiments were conducted to understand the thermal decomposition and melting phenomena of the crystals. The crystal was brown at 300 K, as shown in the inset of Fig. 2. While no changes were observed from 300 to 500 K, the crystal began to melt slightly and changed from brown to dark brown at approximately 543 K. The color change was likely due to decomposition from the loss of HBr, and also due to the geometrical changes in CuBr_4_. Above 600 K, the surface and edges melted significantly. The thermogram clearly indicated that 543 K was the melting point of the crystal. Hence, the [NH_3_(CH_2_)_3_NH_3_]CuBr_4_ crystal is suitable for applications up to 504 K.

### ^1^H NMR chemical shifts and spin–lattice relaxation times

**Figure 3:**
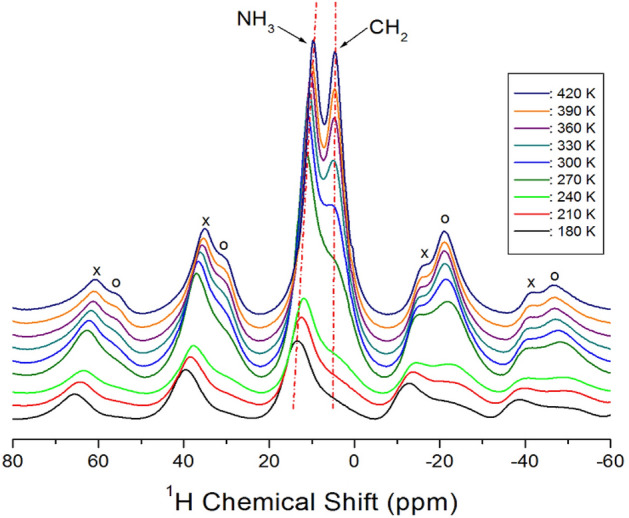
^1^H NMR chemical shifts for NH_3_ and CH_2_ in [NH_3_(CH_2_)_3_NH_3_]CuBr_4_ at several temperatures.

The ^1^H NMR chemical shifts of the [NH_3_(CH_2_)_3_NH_3_]CuBr_4_ crystals were recorded while increasing the temperature, as shown in Fig. 3. Below 270 K, only one ^1^H resonance signal was observed, and the intensity and line-width of the ^1^H signal were very small and wide, respectively, making the detection challenging. The resonance signal exhibited asymmetric shapes owing to the overlapping of the two types of NH_3_ and CH_2_ signals. The ^1^H NMR chemical shift for CH_2_ was recorded at δ = 5.51 ppm at 300 K, whereas that for NH_3_ was recorded at δ = 10.94 ppm, which subsequently split into two resonance lines. The spinning sidebands for CH_2_ are represented by open circles and those for NH_3_ are represented by crosses. The ^1^H chemical shifts for CH_2_, shown by the dotted line in Fig. 3, did not significantly change with increasing temperature, whereas the change in the ^1^H chemical shifts for NH_3_ toward the lower chemical shift as the temperature increased. The greater shift in the ^1^H NMR chemical shift of the NH_3_ in the cation with changes in the temperature, than that of the CH_2_ is reason for the large change in the N−H∙∙∙Br hydrogen bonding between the Br around Cu and the H of NH_3_.

**Figure 4:**
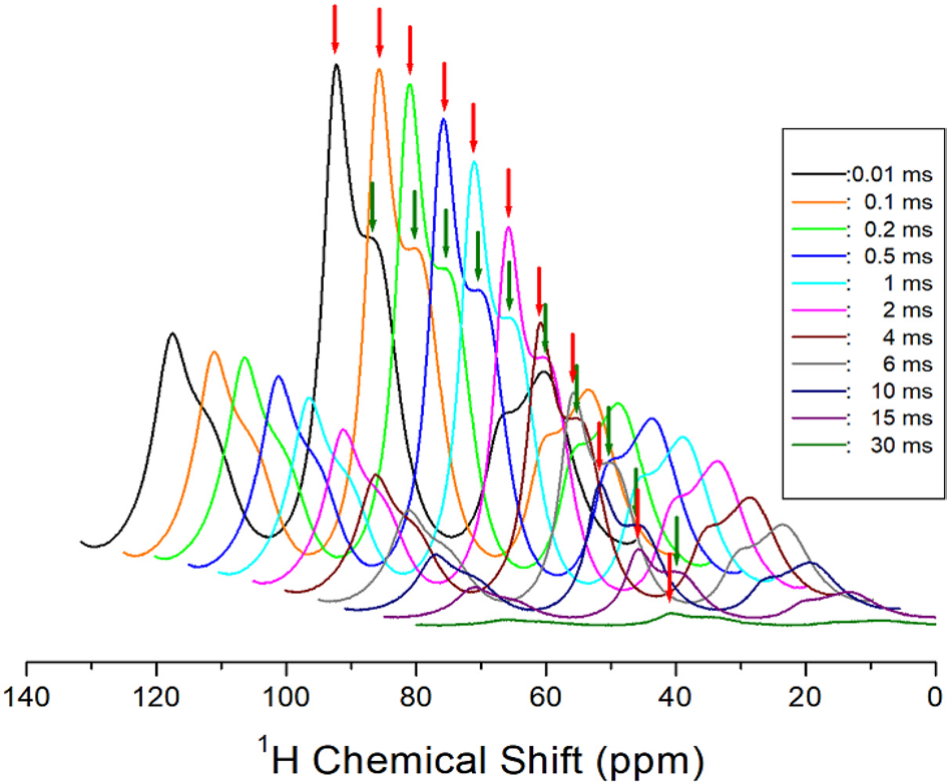
Inversion recovery traces for ^1^H NMR chemical shifts according to the delay time from 0.01 to 30 ms at 300 K.

**Figure 5:**
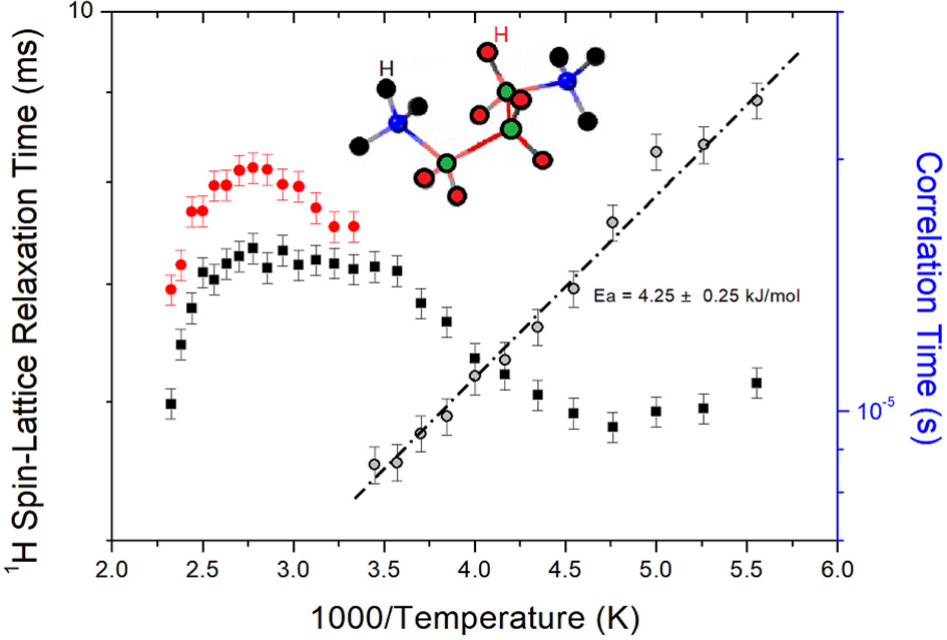
^1^H NMR spin–lattice relaxation times T_1ρ_ and correlation times for NH_3_ and CH_2_ in [NH_3_(CH_2_)_3_NH_3_]CuBr_4_ as a function of inverse temperature.

The relationship of the decay rate of proton magnetization is defined by T_1ρ_ and the Eq. (1)^27,36–39^

1 $$ {\text{P}}(\tau ) = {\text{P}}(0){\text{exp}}( - \tau /{\text{T}}_{{{1}\uprho }} ), $$

where P(*τ*) and P(0) are the signal intensities at times *τ* and *τ* = 0, respectively. The intensity changes observed in the ^1^H NMR spectra were recorded with changing delay times at a given temperature, and at 300 K, the ^1^H NMR spectrum was plotted with a delay time ranging from 0.01 to 30 ms as shown in Fig. 4. From the slope of the intensities of the ^1^H signal indicated by the arrow vs. delay times, the ^1^H T_1ρ_ could be calculated using Eq. (1). As a result, the ^1^H T_1ρ_ values for CH_2_ and NH_3_ are shown in Fig. 5 as a function of the inverse temperature. The ^1^H T_1ρ_ values were in the order of a few milliseconds for CH_2_ and NH_3_, and their values were temperature-dependent. As shown in the cation structure in Fig. 5, the ^1^H of CH_2_ is expressed in red, and the ^1^H of NH_3_ is expressed in black, which is the same as the T_1ρ_ values. The T_1ρ_ values decreased as the temperature increased and then increased sharply again at 210 K. Below 300 K, only the ^1^H T_1ρ_ value for NH_3_ is shown, and the ^1^H T_1ρ_ values of CH_2_ above 300 K have longer T_1ρ_ values than ^1^H of NH_3_. The T_1ρ_ vs. inverse temperature curve showed a minima of 5.80 ms at 210 K, which indicates the existence of distinct molecular motions. The T_1ρ_ values can be explained by the correlation time τ_C_ for the molecular motion. The T_1ρ_ value for the molecular motion based on the Bloembergen–Purcell–Pound (BPP) theory is given by^35–39^:

2 $$ {1}/{\text{T}}_{{{1}\uprho }} = {\text{G}}(\gamma_{{\text{H}}} \gamma_{{\text{C}}} \hbar /r^{{3}} )^{{2}} \left[ {{\text{4F}}_{{\text{a}}} + {\text{F}}_{{\text{b}}} + {\text{3F}}_{{\text{c}}} + {\text{6F}}_{{\text{d}}} + {\text{6F}}_{{\text{e}}} } \right] $$

where F_a_ = τ_C_/[1 + ω_1_^2^τ_C_^2^], F_b_ = τ_C_/[1 + (ω_C_ − ω_H_)^2^τ_C_^2^], F_c_ = τ_C_/[1 + ω_C_^2^τ_C_^2^], F_d_ = τ_C_/[1 + (ω_C_ + ω_H_)^2^τ_C_^2^], and F_e_ = τ_C_/[1 + ω_H_^2^τ_C_^2^]. Here, G is a coefficient, γ_H_ and γ_C_ are the gyromagnetic ratios of the proton and carbon, respectively, ħ is the reduced Planck constant, ω_H_ and ω_C_ are the Larmor frequencies of ^1^H and ^13^C, respectively, *r* is the distance between the proton and carbon, and ω_1_ is the spin-locking pulse sequence with a locking pulse of 71.42 kHz. In the rotating frame, τ_C_ can be obtained when ω_1_τ_C_ = 1, and the coefficient G in Eq. (2) can be obtained from T_1ρ_, ω_H,_ ω_C_, and ω_1_. Using this G value, τ_C_ was obtained as a function of inverse temperature. The local field fluctuation is governed by the thermal motion, which is activated by thermal energy. Therefore, τ_C_ is represented by the Arrhenius behavior: τ_C_ = τ_o_exp(− E_a_/k_B_T), where E_a_ and k_B_ are the activation energy and Boltzmann constant, respectively^36^. The τ_C_ vs. 1000/T was plotted on a logarithmic scale (inset of Fig. 5), and the E_a_ of ^1^H, depending on the molecular dynamics, was obtained using 4.25 ± 0.25 kJ/mol by dot line.

### ^13^C NMR chemical shifts and spin–lattice relaxation times

**Figure 6:**
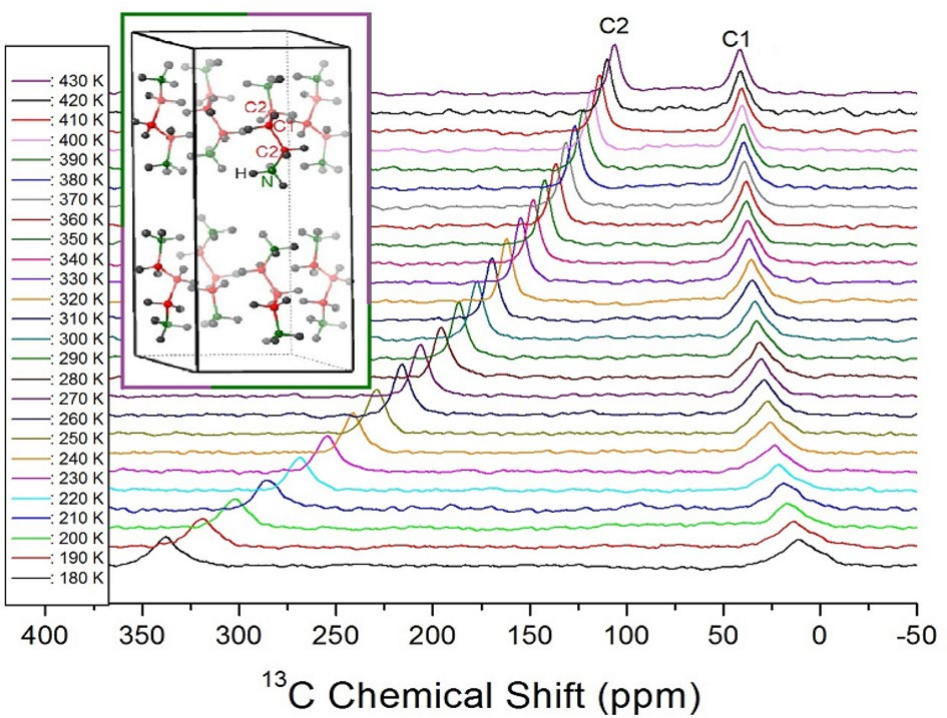
^13^C NMR chemical shifts in [NH_3_(CH_2_)_3_NH_3_]CuBr_4_ as a function of temperature (Inset: structure of [NH_3_(CH_2_)_3_NH_3_] cation).

The ^13^C NMR chemical shifts of [NH_3_(CH_2_)_3_NH_3_]CuBr_4_ were measured as a function of temperature, as shown in Fig. 6. The ^13^C MAS NMR spectra exhibited two resonance signals at all temperatures. The ^13^C NMR spectrum for TMS was recorded at 38.3 ppm at 300 K and was used to determine the exact chemical shift of ^13^C^27^. Here, the CH_2_ between the two CH_2_ groups is labeled C1, and the CH_2_ close to NH_3_ is labeled C2, as shown in the inset of Fig. 6. The two resonance signals at 300 K were recorded at chemical shifts of δ = 33.54 and δ = 177.07 ppm for C1 and C2, respectively. The ^13^C chemical shifts for CH_2_ were different for C1 far from those of NH_3_ and C2 close to that of NH_3_. The ^13^C chemical shift for C1 changed slowly and did not vary significantly with increasing temperature, whereas that for C2 moved abruptly to the lower chemical-shift side with increasing temperature compared to that for C1.

**Figure 7:**
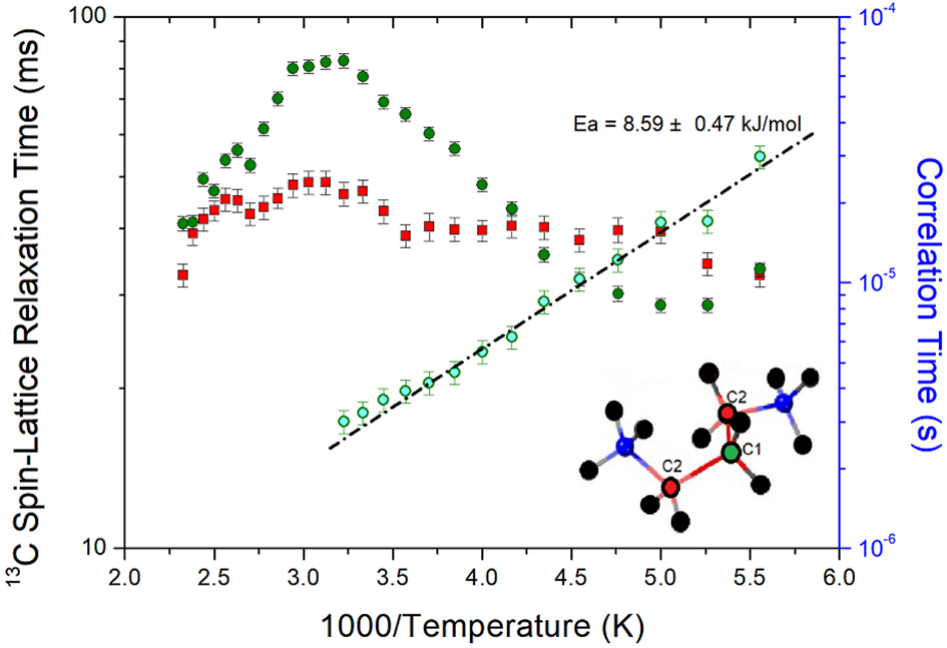
^13^C NMR spin–lattice relaxation times T_1ρ_ and correlation times for C1 and C2 of [NH_3_(CH_2_)_3_NH_3_]CuBr_4_ as a function of inverse temperature.

The changes in the intensities of the ^13^C NMR spectral peaks with increasing delay times were measured at a given temperature in the same manner as the ^1^H T_1ρ_ measurement method. The ^13^C T_1ρ_ values from the slope of the recovery traces are described by a single exponential function in Eq. (1). The T_1ρ_ values for C1 and C2 as a function of 1000/temperature are shown in Fig. 7. In the cation structure, C1 is shown in green, C2 is shown in red, and T_1ρ_ is shown in the same way. ^13^C T_1ρ_ values for C2 showed no changes in the temperature range measured in this study, and the T_1ρ_ for C1 according to the temperature change showed a similar trend as that for ^1^H T_1ρ_. The ^13^C T_1ρ_ values were approximately 10 times longer than the ^1^H T_1ρ_ values. The ^13^C T_1ρ_ values were unaffected by the spin diffusion owing to the small dipolar coupling, which results from the low natural abundance. On the other hand, the T_1ρ_ values decreased as the temperature increased, and then increased again at 200 K. Below 300 K, only the ^13^C T_1ρ_ value for C1 vs. the inverse temperature showed a minimum of 28.58 ms at 200 K, which implies the existence of active molecular motions at low temperatures. τ_C_ values on a logarithmic scale, as obtained by Eq. (2) vs. 1000/T, were plotted (inset of Fig. 7). The E_a_, depending on the molecular dynamics of ^13^C, was measured to be 8.59 ± 0.47 kJ/mol. The ^13^C E_a_ value was approximately twice that of ^1^H E_a_.

## Discussion

^1^H and ^13^C NMR T_1ρ_ values in [NH_3_(CH_2_)_3_NH_3_]*Me*Br_4_ (*Me*=Cu, Zn, and Cd) crystals were compared and discussed with previously reported results^27,35^. In single crystals of [NH_3_(CH_2_)_3_NH_3_]*Me*Br_4_ (*Me*=Cu, Zn, and Cd), the crystal structure, phase transition temperature, and T_1ρ_ for ^1^H and ^13^C when the cation lengths are the same and the *Me* is different, are shown in Table 1. The changes in the chemical shifts of both ^1^H and ^13^C for three crystals with temperature are shown in Supplementary Information 2 and 3. When Zn and Cd were included, the ^1^H and ^13^C chemical shifts were similar, whereas when Cu was included, the chemical shifts were different. The differences in the chemical shifts are due to the differences in the electron structures of the metal ions, *Me*. Cu^2+^ has one electron, outside the closed *d-*shell. Zn^2+^ and Cd^2+^ have two electrons outside its closed shell^37^.

**Table 1:** Structure, lattice constants, phase transition temperature T_C_, and spin–lattice relaxation time T_1ρ_ of [NH_3_(CH_2_)_3_NH_3_]*Me*Br_4_ (*Me* = Cu, Zn, and Cd) crystals.

*Me*	Cu	Zn	Cd
Structure	Monoclinic	Monoclinic	Orthorhombic
Lattice constants (Å)	a = 8.086	a = 11.084	a = 7.898
	b = 7.566	b = 10.968	b = 7.721
	c = 17.622	c = 11.185	c = 19.054
	β = 96.75	β = 117.07	
T_C_ (K)	x	272	326, 368
^1^H T_1ρ_ (ms) at 300 K	7.14 (for NH_3_)	236.38	280.16
	7.55 (for CH_2_)		
^13^C T_1ρ_ (ms)at 300 K	77.19 (for C1)	6.80 (for C1)	81.59 (for C1)
	47.00 (for C2)	5.86 (for C2)	59.79 (for C2)

**Figure 8:**
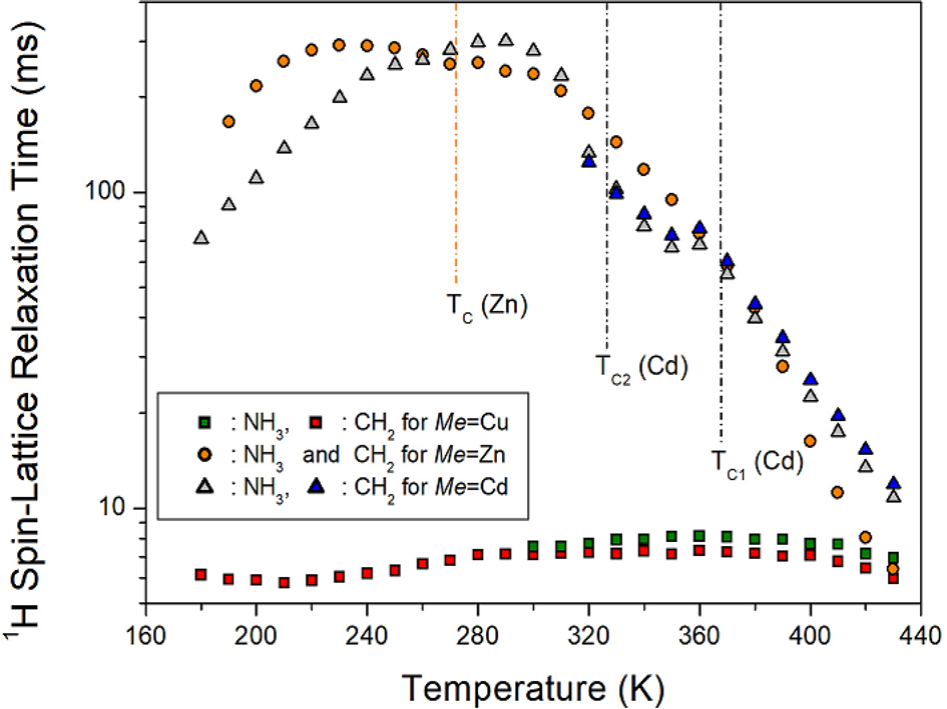
^1^H NMR spin–lattice relaxation times T_1ρ_ of [NH_3_(CH_2_)_3_NH_3_]*Me*Br_4_ (*Me* = Cu, Zn, and Cd) as a function of temperature.

**Figure 9:**
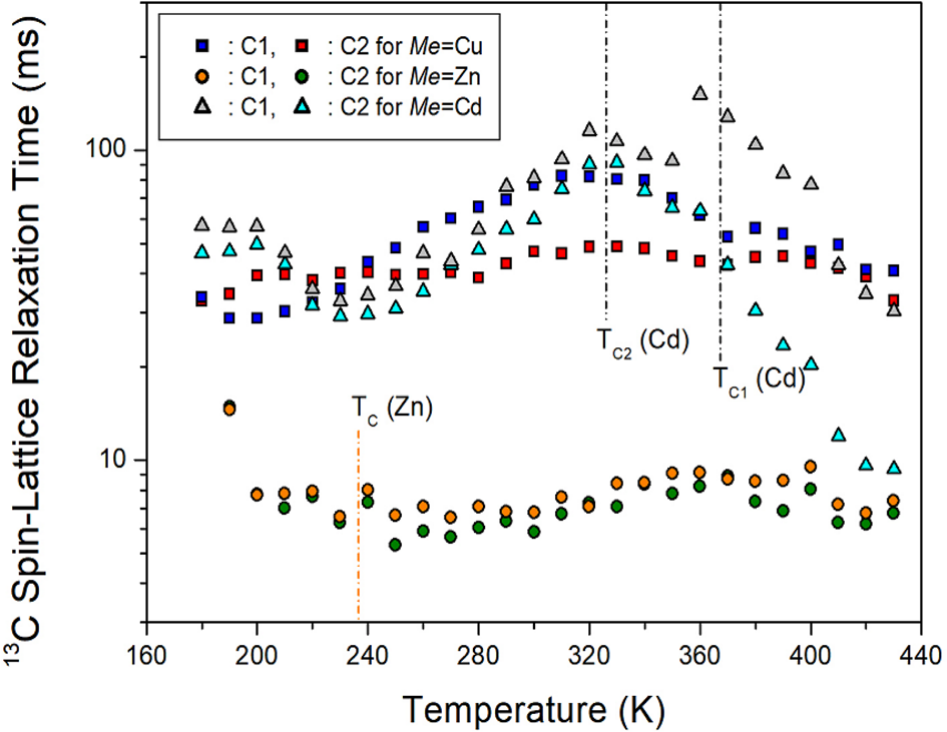
^13^C NMR spin–lattice relaxation times T_1ρ_ of [NH_3_(CH_2_)_3_NH_3_]*Me*Br_4_ (*Me* = Cu, Zn, and Cd) as a function of temperature.

^1^H T_1ρ_ values with a paramagnetic Cu^2+^ ion have a very different trend from those of Zn^2+^ and Cd^2+^ without the paramagnetic ion; Zn and Cd show a very similar trend and indicate temperature dependence alone (see Fig. 8). T_1ρ_ has a very less value because of the Cu^2+^-containing paramagnetic ions. Because T_1ρ_ is inversely proportional to the square of the magnetic moment of the paramagnetic Cu^2+^ ions, the value is in the order of a few milliseconds. Unlike the ^1^H T_1ρ_ values, the ^13^C T_1ρ_ values when Zn is included are in the order of 10 ms, and the ^13^C T_1ρ_ values when Cu and Cd are included have a very long order of 100 ms, and are sensitive to temperature, as shown in Fig. 9. From these results, the effect of paramagnetic ions was directly affected by ^1^H, which is close to that of Cu, but ^13^C T_1ρ_ was not significantly affected by the paramagnetic ions. In addition, the structures of Cu and Cd are related to the octahedral *Me*Br_6_, and the structure of Zn is related to tetrahedral *Me*Br_4_. The ^13^C T_1ρ_ values in [NH_3_(CH_2_)_3_NH_3_]CuBr_4_ and [NH_3_(CH_2_)_3_NH_3_]CdBr_4_ with the two-dimensional octahedral *Me*Br_6_ structure have longer values than those in [NH_3_(CH_2_)_3_NH_3_]ZnBr_4_ with the zero-dimensional structure of the tetrahedral *Me*Br_4_. The ^1^H T_1ρ_ is affected by the paramagnetic ion of the cation, and the ^13^C T_1ρ_ is thought to be affected by the *MeX*_4_ or *MeX*_6_ structure of the anion.

## Conclusion

To investigate the structural, thermal, and physical properties of the lead-free hybrid perovskite [NH_3_(CH_2_)_3_NH_3_]CuBr_4_ crystal, we performed XRD, DSC, TGA, optical polarizing microscopy, and NMR spectroscopy. First, the monoclinic structure of this crystal was confirmed by XRD, and its thermodynamic stability was observed at approximately 500 K without phase transition. Second, the NMR chemical shifts were due to the local field around the resonating nucleus in the crystals, and the chemical shifts in the ^1^H and ^13^C NMR spectra indicated changes in the crystallographic geometry. The ^1^H chemical shifts of NH_3_ changed significantly with temperature compared to those of the CH_2_ because the structural environment around the ^1^H of NH_3_ changed to a greater degree than the structural environment around the ^1^H of CH_2_. In addition, the ^13^C chemical shifts for C1 increased slowly with increasing temperature, whereas the chemical shifts for C2 shifted significantly compared to C1. Consequently, the large changes in the ^1^H chemical shifts of NH_3_ and the chemical shift of C2 close to N were affected by the N−H∙∙∙Br hydrogen bonds owing to the extensive changes in the structural geometry of CuBr_4_. Finally, ^1^H and ^13^C T_1ρ_ showed very similar trends for temperature changes; however, the ^1^H T_1ρ_ values were approximately 10 times shorter than the ^13^C T_1ρ_ values. T_1ρ_ values show that the energy transfer, with a large thermal displacement around the ^1^H atoms of the cation, is very short. E_a_ values for ^1^H and ^13^C at low temperatures were 4.25 and 8.59 kJ/mol, respectively, indicating that the value for ^1^H was smaller.

The detailed elucidation of these physical properties is expected to greatly facilitate the development of potential eco-friendly material applications. In the future, we plan to study [NH_3_(CH_2_)_3_NH_3_]*Me*I_4_, which may be more suitable candidate for solar cells. Furthermore, as the most popular approaches to materials design, we plan to expand and study a high-quality film and relevant characterizations.

## Supplementary Information

Supplementary Information 1.

Supplementary Information 2.

Supplementary Information 3.

## Data Availability

The datasets generated and/or analysed during the current study are available from the corresponding author on reasonable request.
