# Supplementary figures and images for: Advances in physicochemical characterization of lead-free hybrid perovskite [NH3(CH2)3NH3]CuBr4 crystals
Source: Sci Rep. 2022 May 24;12:8769. doi: 10.1038/s41598-022-12832-y (PMC9130295; doi:10.1038/s41598-022-12832-y)

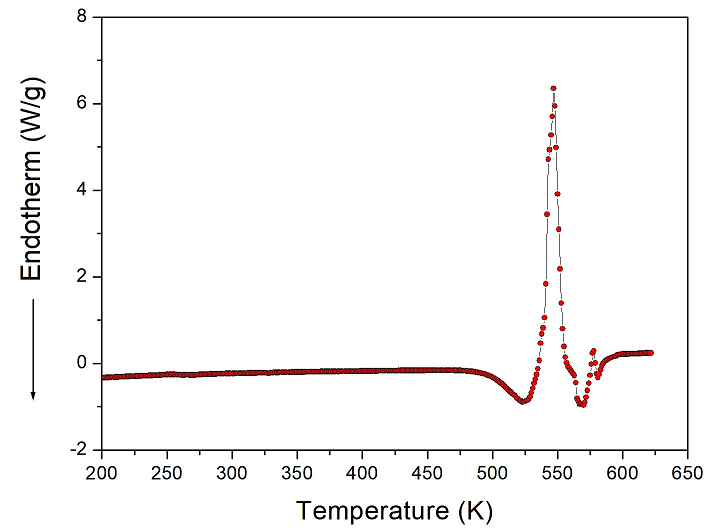


**Figure** Differential scanning calorimetry (DSC) thermogram of [NH3(CH2)3NH3]CuBr4.

Supplement: Supplementary file 1 — Supplementary Information 1. [file 41598_2022_12832_MOESM1_ESM.docx]

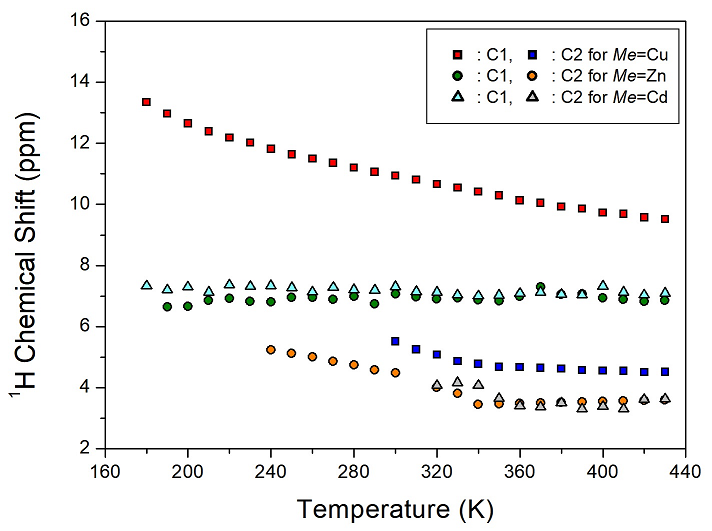


**Figure** 1H NMR chemical shifts for NH3 and CH2 in [NH3(CH2)3NH3]*Me*Br4 (*Me*=Cu, Zn, Cd)as a function of temperature.

Supplement: Supplementary file 2 — Supplementary Information 2. [file 41598_2022_12832_MOESM2_ESM.docx]

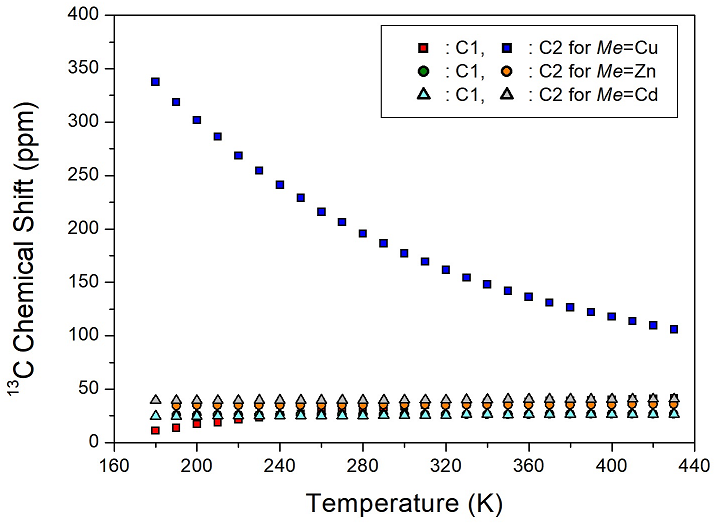


**Figure** 13C NMR chemical shifts for C1 and C2 in [NH3(CH2)3NH3]*Me*Br4 (*Me*=Cu, Zn, Cd)as a function of temperature.

Supplement: Supplementary file 3 — Supplementary Information 3. [file 41598_2022_12832_MOESM3_ESM.docx]
